# Gradient washout and secondary nephrogenic diabetes insipidus after brain injury in an infant: a case report

**DOI:** 10.1186/s13256-020-02536-0

**Published:** 2020-10-10

**Authors:** Nathan Chang, Karley Mariano, Lakshmi Ganesan, Holly Cooper, Kevin Kuo

**Affiliations:** 1 Department of Pediatric Critical Care Medicine, Lucile Packard Children’s Hospital Stanford, Palo Alto, CA USA; 2 Department of Pediatric Nephrology, Lucile Packard Children’s Hospital Stanford, Palo Alto, CA USA; 3 Department of Pediatric Endocrinology, Lucile Packard Children’s Hospital Stanford, Palo Alto, CA USA

**Keywords:** Gradient washout, Nephrogenic diabetes insipidus, Central diabetes insipidus, Cerebral salt wasting, Brain injury

## Abstract

**Background:**

Disorders of water and sodium balance can occur after brain injury. Prolonged polyuria resulting from central diabetes insipidus and cerebral salt wasting complicated by gradient washout and a type of secondary nephrogenic diabetes insipidus, however, has not been described previously, to the best of our knowledge. We report an unusual case of an infant with glioblastoma who, after tumor resection, was treated for concurrent central diabetes insipidus and cerebral salt wasting complicated by secondary nephrogenic diabetes insipidus.

**Case presentation:**

A 5-month-old Hispanic girl was found to have a large, hemorrhagic, suprasellar glioblastoma causing obstructive hydrocephalus. Prior to mass resection, she developed central diabetes insipidus. Postoperatively, she continued to have central diabetes insipidus and concurrent cerebral salt wasting soon after. She was managed with a vasopressin infusion, sodium supplementation, fludrocortisone, and urine output replacements. Despite resolution of her other major medical issues, she remained in the pediatric intensive care unit for continual and aggressive management of water and sodium derangements. Starting on postoperative day 18, her polyuria began increasing dramatically and did not abate with increasing vasopressin. Nephrology was consulted. Her blood urea nitrogen was undetectable during this time, and it was thought that she may have developed a depletion of inner medullary urea and osmotic gradient: a “gradient washout.” Supplemental dietary protein was added to her enteral nutrition, and her fluid intake was decreased. Within 4 days, her blood urea nitrogen increased, and her vasopressin and fluid replacement requirements significantly decreased. She was transitioned soon thereafter to subcutaneous desmopressin and transferred out of the pediatric intensive care unit.

**Conclusions:**

Gradient washout has not been widely reported in humans, although it has been observed in the mammalian kidneys after prolonged polyuria. Although not a problem with aquaporin protein expression or production, gradient washout causes a different type of secondary nephrogenic diabetes insipidus because the absence of a medullary gradient impairs water reabsorption. We report a case of an infant who developed complex water and sodium imbalances after brain injury. Prolonged polyuria resulting from both water and solute diuresis with low enteral protein intake was thought to cause a urea gradient washout and secondary nephrogenic diabetes insipidus. The restriction of fluid replacements and supplementation of enteral protein appeared adequate to restore the renal osmotic gradient and efficacy of vasopressin.

## Background

Central disorders of water and sodium balance are known complications after brain injury. Insult surrounding the hypothalamus and pituitary gland increases the risk for antidiuretic hormone (ADH) dysregulation, causing transient or permanent central diabetes insipidus (CDI) and, in some cases, syndrome of inappropriate antidiuretic hormone (SIADH) [1, 2]. Brain injury has also been associated with a cerebral salt wasting syndrome (CSW) that can occur with CDI, which is treated with fluid and sodium replacement [3, 4]. We report a case of combined CDI and CSW refractory to conventional treatment. This was ultimately thought to have occurred because of gradient washout with secondary nephrogenic diabetes insipidus (NDI). Gradient washout is a depletion of interstitial medullary sodium or urea that reduces water reabsorption, despite the presence of functioning ADH. The patient responded quickly to protein supplementation and fluid restriction, supporting this diagnosis. To our knowledge, this is the first report of combined CDI and CSW complicated by secondary NDI.

## Case presentation

We present a case of a 6.8-kg, 5-month-old Hispanic girl who was brought to the emergency room for 1 day of fever, vomiting, seizures, and leftward gaze deviation. Initial computed tomography revealed a large hemorrhagic intracranial mass in the suprasellar and third ventricular region causing obstructive hydrocephalus. An emergent extraventricular drain was placed, a magnetic resonance imaging study was obtained, and the girl was admitted to the pediatric intensive care unit (PICU). Other than left gaze preference and clonus, the result of her examination was unremarkable, with normal capillary refill and no sunken fontanels, though a full examination was limited by sedation and her hydrocephalus. On hospital day 3, the mass was surgically resected, and its pathology was consistent with stage IV glioblastoma.

**Fig. 1:**
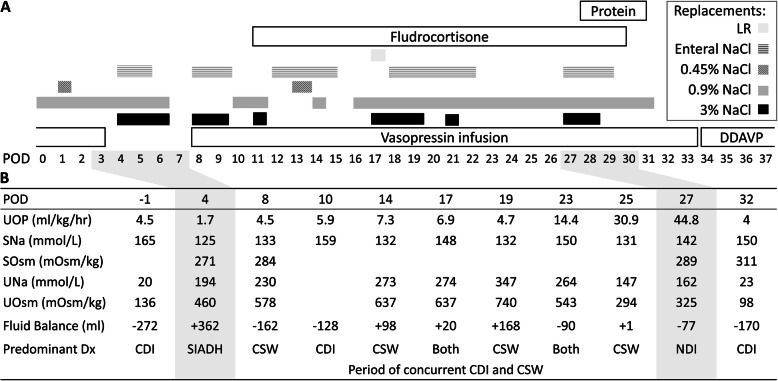
Treatment and laboratory data by postoperative day (POD). **a** Treatments by time. When CDI was predominant, vasopressin was titrated. When CSW was predominant, sodium supplementation was prioritized. For fluid losses from both diagnoses, on most days, 0.9% NaCl was the chosen replacement. The period of nephrogenic diabetes insipidus was treated with reduction in fluid replacement and enteral protein supplement. **b** Select examples of diagnostic laboratory results and the predominant diagnosis that guided treatment at that time. *CDI* Central diabetes insipidus, *CSW* Cerebral salt wasting, *DDAVP* Subcutaneous desmopressin, *Dx* Diagnosis, *Fluid balance* Documented fluid balance over a 24-hour period, *LR* Lactated Ringer solution, *SIADH* Syndrome of inappropriate antidiuretic hormone, *SNa* Serum sodium concentration, *SOsm* Serum osmolality, *UNa* Urine sodium concentration, *UOP* Urine output, *UOsm* Urine osmolality

Prior to resection, she developed CDI, and a vasopressin infusion was initiated. Vasopressin was titrated to maintain urine output between 2 and 4 ml/kg/hour and was discontinued on postoperative day (POD) 1. On POD 3, she became hyponatremic with low to normal urine output, thought to be SIADH. On POD 7, she again became polyuric, but with continued hyponatremia and elevated urine sodium. She was started on sodium and fluid supplementation in the form of 0.9% NaCl, 3% NaCl, and enteral NaCl tablets to treat CSW. On POD 8, she developed brisk and dilute urine output, with serum hypernatremia, requiring reinitiation of a vasopressin infusion and temporary cessation of sodium supplementation for CDI (Fig. 1). By the next day, she became hyponatremic with continued polyuria, suggestive of concurrent ongoing CSW, and sodium supplementation was resumed.

She fluctuated between hypo-osmolar and hyperosmolar polyuria. Trials of discontinuing vasopressin only resulted in worsened polyuria, so she remained on a vasopressin infusion. To promote renal sodium retention, fludrocortisone was started at 0.025 mg twice daily on POD 11 and increased to three times daily on POD 17 [4, 5]. Sodium supplementation was adjusted to maintain normonatremia. Urine output in excess of 4 ml/kg/hour was replaced with lactated Ringer solution, 0.45% NaCl, and 0.9% NaCl respective of her urine sodium content. A timeline of various fluid and sodium treatments and examples of laboratory findings that guided treatment are described in Fig. 1. It was presumed that the component CSW would resolve over time. Her serum sodium concentration remained generally stable, with urine output ranging from 2 to 8 ml/kg/hour. Her fluid balance was nearly even, although maintaining this balance required aggressive and frequent fluid replacement.

**Fig. 2:**
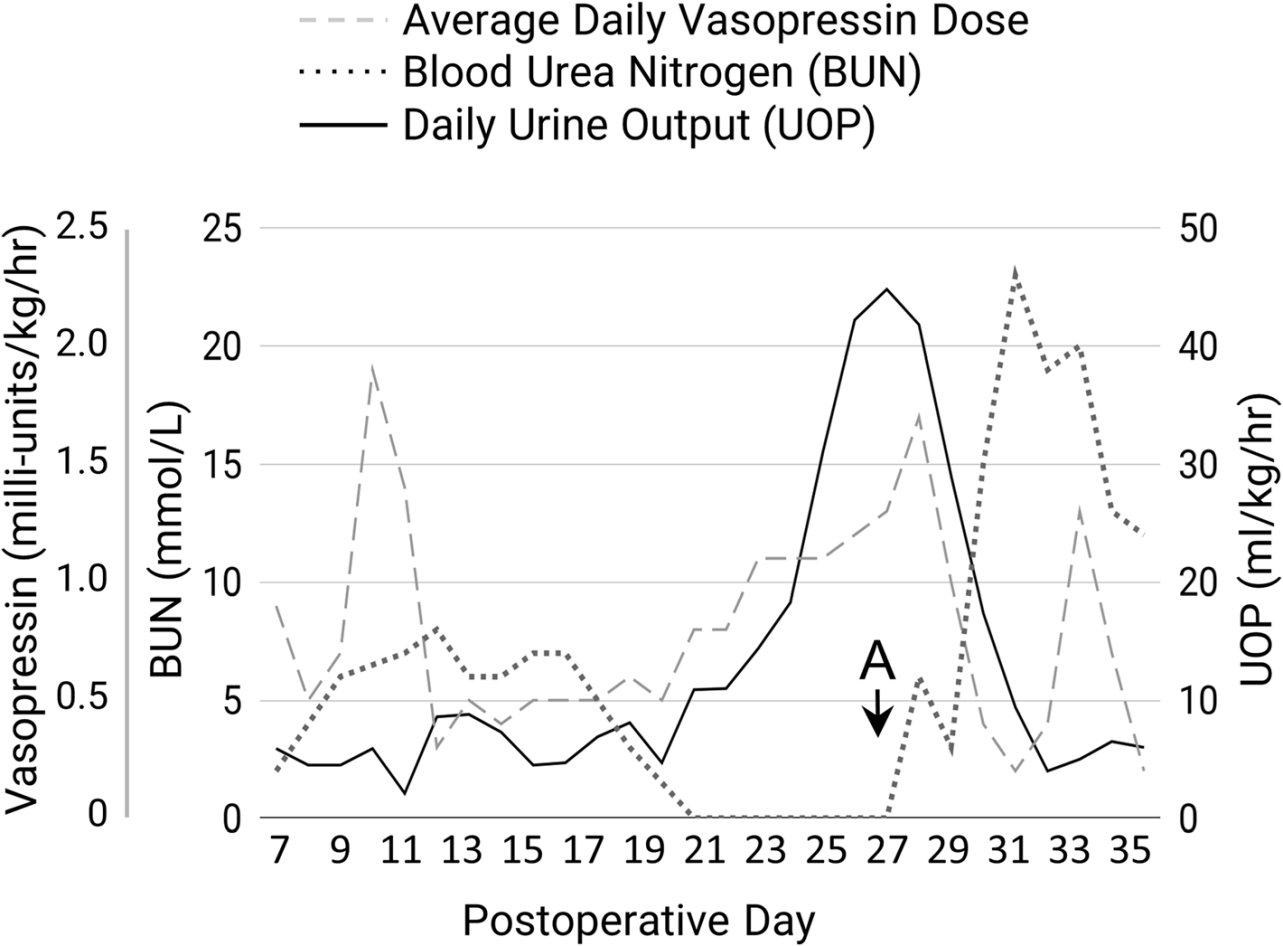
Vasopressin dose, blood urea nitrogen (BUN), and urine output (UOP) over time. Vasopressin dose is reported as average hourly dose per day. Urine output is reported as average hourly output per day. Time point A is initiation of dietary protein supplementation

On POD 20, her urine output began increasing precipitously, reaching over 40 ml/kg/hour in the following days. Her vasopressin infusion was increased accordingly, with no effect in slowing her output (Fig. 2). By this time, her acute neurosurgical issues had otherwise resolved, but she remained in the PICU for fluid management and vasopressin. Her fluid volume status was again maintained, with moist mucous membranes and flat fontanels observed on examination, though she required progressively larger fluid replacement to match her rising urine output. This pronounced polyuria, and apparent inefficacy of increased vasopressin prompted consultation with pediatric nephrology on POD 27.

She had previously responded well to vasopressin; she was given no known medications associated with NDI; her electrolytes other than sodium and chloride were within normal limits; and her creatinine was appropriate for her age, ranging from 0.06 to 0.12 mg/dl, ruling out most acute and congenital causes of NDI. Other causes of urinary concentration defects were therefore considered. Her total protein upon admission was normal, but her serum albumin had decreased from 3.7 mg/dl upon admission to 2.9 mg/dl. In the days prior to consultation, her hemoglobin decreased from 11.5 to 8.5 g/dl. Her blood urea nitrogen (BUN) became undetectable, which was thought to reflect a protein deficiency and depleted renal urea gradient (Fig. 2). Prolonged increased fluid and solute intake with hypertension and daily sodium intake of 20 mEq/kg/day were also thought to exacerbate her polyuria, further depleting her osmotic gradient. As such, her fluid replacement was incrementally decreased by 2 ml/kg/hour each day, and she was started on enteral protein supplementation to restore her osmotic gradient. Within 4 days of treatment, her BUN increased, and her urine output, vasopressin needs, and fluid replacement dramatically decreased (Fig. 2). Fludrocortisone was discontinued; protein supplementation was stopped by POD 31. She then exhibited a singular CDI physiology and remained only on vasopressin without need for frequent fluid replacements. She was transitioned to subcutaneous desmopressin on POD 33 and transferred to the general ward on POD 39. After hospital discharge, she remained on desmopressin long term.

## Discussion

We present a case of an infant who experienced significant brain injury followed by complex sodium and water imbalances. These imbalances were refractory to conventional treatment for CDI and CSW and led to a gradient washout that exacerbated these derangements. Enteral protein supplementation and small reductions in replacement fluids were adequate to treat the patient’s marked polyuria, with return of vasopressin efficacy within days. Clinicians treating such patients should recognize this potential phenomenon of gradient washout and secondary NDI.

Central disorders of water balance are anticipated in children with suprasellar masses [1]. Combined CDI and CSW, though, is uncommon, with limited reports describing their concurrence [6–8]. CSW is generally transient but can last for weeks to months. Treatment of combined CDI and CSW can be difficult. Sodium supplementation and fluid resuscitation are reported in all cases, and fludrocortisone may have benefit in some [3–8]. The role of ADH in these cases is unclear. Some authors recommend the continued use of vasopressin, whereas others suggest that natriuresis can be resistant to and even aggravated by exogenous ADH [7–9]. In our patient, the abrupt onset of worsening polyuria after a previous period of ADH responsiveness suggested a nephrogenic pathology beyond the effects of ADH in treating combined CDI and CSW alone.

**Fig. 3:**
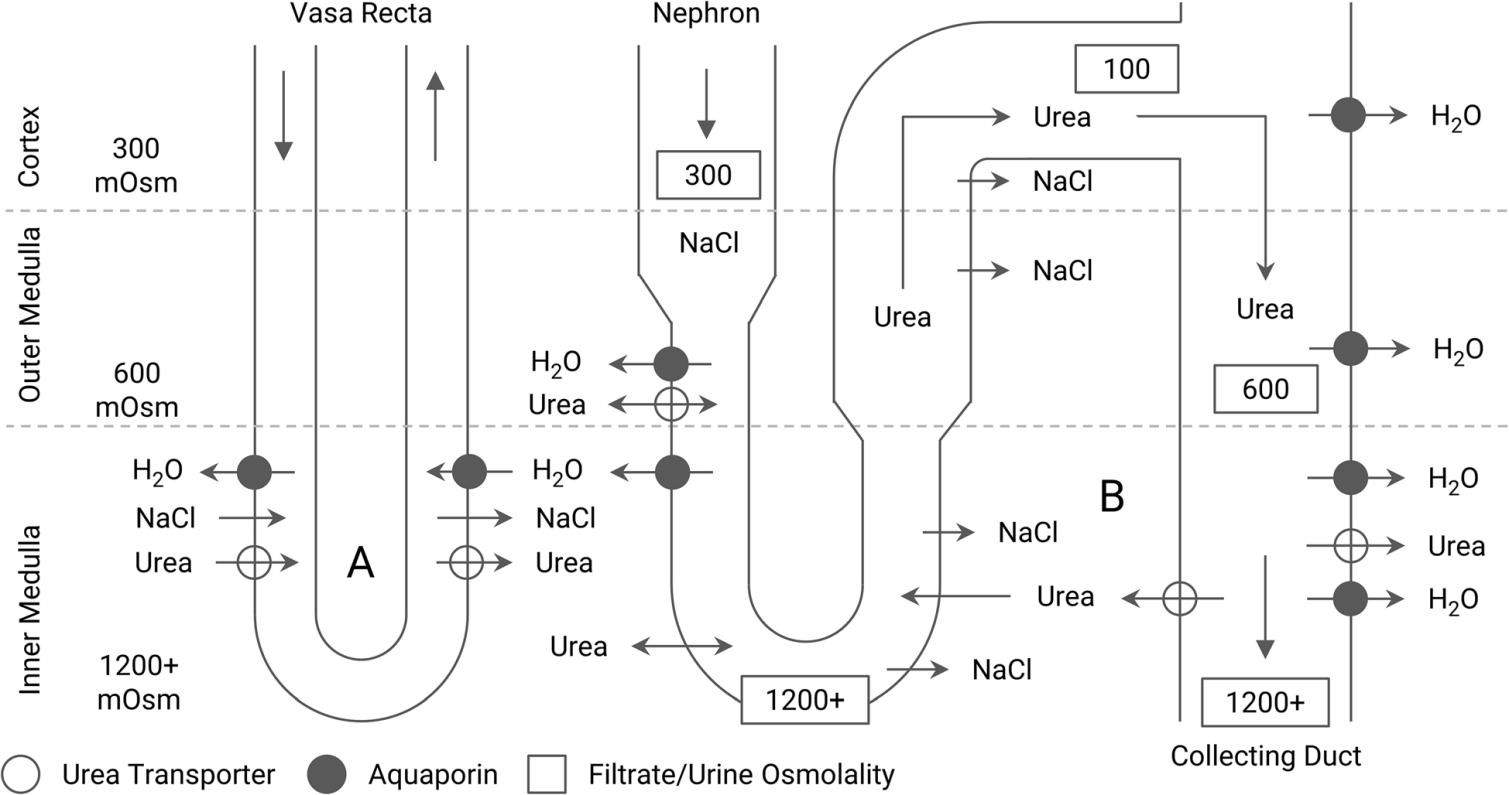
Renal concentrating mechanism and the movement of urea. **a** Inner medullary countercurrent exchange through the descending and ascending vasa recta. **b** Urea recycling from the inner medullary collecting duct

Mammalian kidneys maintain an osmotic gradient from the corticomedullary boundary to the inner medulla that is required for passive reabsorption of free water when ADH is present [10]. The gradient is sustained by the reabsorption of NaCl in the outer medulla and NaCl with urea in the inner medulla (Fig. 3). ADH also supports this gradient by activating NaCl and urea transporters. Urea recycling in the collecting duct relies on passive and active transport and is affected by urinary transit time and tubular osmolality [10]. Excessive and dilute urinary flow, therefore, can impair urea reabsorption. High blood flow through the vasa recta can also disturb the natural countercurrent exchange responsible for maintaining medullary concentration. Without recycling of urea from the collecting duct and without systemic delivery of urea to the inner medulla, a gradient washout, or depletion of the osmotic gradient, can occur [11, 12]. Without a gradient, ADH is ineffective. Although not an inherent problem with aquaporin function, expression, or production, gradient washout is considered a type of secondary NDI [13].

Prolonged polydipsia and polyuria leading to impaired urinary concentrating ability has long been observed in animals and humans. Chronically polydipsic rats exhibited hypo-osmolar polyuria that persisted despite having increased ADH levels during water deprivation [14]. Human adults, after a period of high water intake, experienced a transient decreased ability to concentrate urine during water deprivation [15]. Gradient washout is one of the purported mechanisms explaining this urinary concentration defect. During forced water ingestion, human adults demonstrate both increased urinary urea loss and decreased serum urea, with impaired urinary concentration during subsequent water deprivation [16]. In a human case of suspected psychogenic polydipsia, gradient washout was suggested to have propagated polyuria [17]. These urinary concentration abnormalities improved following a period of water deprivation [15–17]. In the setting of low-protein diets, supplemental urea and protein also can improve urinary concentration by regulating urea transporter expression, enhancing aquaporin protein abundance, and increasing interstitial medullary urea and hypertonicity [18–22].

In our patient, we hypothesize that a prolonged period of polyuria with high fluid intake reduced the ability of her medullary interstitium to resorb urea. This urea gradient washout then caused an apparent inefficacy of vasopressin. She did not initially present with signs of malnutrition, weighing near the 50th percentile for her age and with normal protein markers upon admission. The protein content of breast milk, however, was likely inadequate for her acute high-output state, reducing the availability of urea to replenish the medullary concentration. High solute load with NaCl may also have affected vasa recta flow and countercurrent exchange. Therapeutically, a relative water restriction by way of reduced urine output replacement was started with nutritional protein supplementation. These interventions appeared to have a direct temporal relationship in reversing her extreme polyuria by restoring urea balance and, among other benefits noted earlier, reestablishing her medullary gradient.

## Conclusion

Gradient washout has not been widely reported in humans. We report a case of an infant who developed complex water and sodium imbalances after brain injury, which, to our knowledge, have not been described previously. Prolonged polyuria from both water and solute diuresis with low enteral protein intake was suspected to cause a urea gradient washout and secondary NDI. Supplementation of enteral protein and reduction in fluid intake were adequate to restore her renal osmotic gradient and the efficacy of vasopressin. This case highlights the critical physiologic role of urea and the interstitial medullary gradient in maintaining water homeostasis. Clinicians should be judicious with fluid replacement volume and consider the possibility of gradient washout and secondary NDI in such patients.

## Data Availability

Data relevant to this case report are included within the article text and figures.

## Abbreviations

ADH: Antidiuretic hormone
CDI: Central diabetes insipidus
CSW: Cerebral salt wasting
DDAVP: Subcutaneous desmopressin
Dx: Diagnosis
LR: Lactated Ringer solution
NDI: Nephrogenic diabetes insipidus
PICU: Pediatric intensive care unit
POD: Postoperative day
SIADH: Syndrome of inappropriate antidiuretic hormone
SNa: Serum sodium concentration
SOsm: Serum osmolality
Una: Urine sodium concentration
UOP: Urine output
UOsm: Urine osmolality
